# Cassava Orange-Fleshed Sweetpotato Composite Gari: A Potential Source of Dietary Vitamin A

**DOI:** 10.3389/fnut.2021.646051

**Published:** 2021-06-07

**Authors:** Richard Atinpoore Atuna, Matthew Atongbiik Achaglinkame, Trudy Abla Sitsofe Accorley, Francis Kweku Amagloh

**Affiliations:** ^1^Department of Food Science and Technology, University for Development Studies, Tamale, Ghana; ^2^Department of Agricultural Mechanization and Irrigation Technology, University for Development Studies, Tamale, Ghana

**Keywords:** carotene, cassava, cyanogenic compound, gari, orange-fleshed sweetpotato, provitamin A

## Abstract

Gari, a fermented granular cassava food product, continues to play a major role in the diets of West Africans. The white cassava commonly used for this product is devoid of provitamin A but may have a significant concentration of cyanogenic compounds. The physicochemical and functional properties of partial substitution of cassava with orange-fleshed sweetpotato (OFSP) to process gari were investigated. Two commonly consumed products “*eba”* and “*soaked gari*” were prepared from the various formulations and sensorially assessed. Cassava OFSP composite gari (77% cassava:23% OFSP, 75% cassava:25% OFSP, and 73% cassava:27% OFSP) did not significantly (*p* > 0.05) influence the moisture content (3.39%−5.42%, *p* = 0.38), water absorption capacity (589–671 mL/g, *p* = 0.22), and swelling index (3.75–4.17, *p* = 0.08) compared with that of 100% cassava gari. Expectedly, increasing the levels of OFSP incorporation significantly (*p* < 0.001) resulted in color change from white (L* = 83.99, a* = 0.93, b* = 16.35) to orange (L* = 69.26, a* = 7.74, b* = 28.62). For β-carotene, the 73% cassava:27% sample was ~5.2 times more than the level in 100% cassava gari. Also, it had lower residual cyanogenic compounds (0.37 vs. 1.71 mg/kg, *p* < 0.001, measured as hydrogen cyanide) compared with cassava-only gari. The sensory scores by the 100 panelists using a five-point hedonic scale (1 = dislike extremely to 5 = like extremely) exceeded the minimum threshold (3) for acceptance. Within the limits of this study, OFSP can be composited with cassava up to 27% to process gari that has similar physicochemical properties and sensorial preference as that of cassava only. Furthermore, the OFSP-composited gari contains a significant amount of provitamin A and have a reduced residual cyanogenic compound. Thus, the composited gari could play a significant role in addressing vitamin A deficiency in Ghana compared to the 100% cassava only.

## Introduction

Gari is one of the most widely and frequently consumed food staples in many parts of Africa, including Ghana (1–3). Gari is a fermented, partly gelatinized, creamy-white grits traditionally made from cassava (*Manihot esculenta* Crantz) roots (4, 5). Its affordability, shelf stability, and ability to quench hunger have gained its great patronage in West Africa, especially among the poor in society (6). Additionally, the convenience and the ready-to-use nature of this product have further broadened its market by attracting students at all levels and the busy in society (4). Gari is reported to constitute close to 70% of all cassava products and to be the most consumed cassava product in Ghana (7, 8). Duah et al. (7) found that the younger age groups (18–39 years) in their study consumed gari most frequently as compared to the older age groups. Gari is a major staple among households and all classes of pupils or students (8), of which children younger than 5 years may be inclusive, particularly those from poor households. Notably, gari is one of the foods used in national institutional feeding menus in Ghana. However, the conventional cassava grits are deficient in antioxidants such as β-carotene (a provitamin A carotenoid), and other non–provitamin A carotenoids, as the widely grown white cassava is deficient in these health-promoting compounds. Again, the raw material for cassava grits, cassava, is a major source of residual cyanogenic compounds, measured as hydrogen cyanide (HCN) (9).

Vitamin A deficiency (VAD) in Ghana is estimated to affect ~20% of children with higher prevalence (31%) in the deprived rural Northern Ghana (10). Although other factors, such as infections, contribute to VAD, poor dietary intake of vitamin A–rich foods is reported to be the leading cause (11, 12). This nutritional cause of VAD is particularly predominant among the poor because of lack of food diversification and unaffordability of some vitamin A–rich foods, such as carrots and liver (11). Therefore, the campaign for food-based diets rich in vitamin A, or its precursor (e.g., β-carotene), is regarded as one of the most sustainable approaches to addressing VAD, as supplementation and fortification are short-term (13, 14). The characteristics of gari as stated above make it a very good candidate for food-to-food fortification as it is a common food consumed by the poor, the most vulnerable to VAD.

Residual cyanogenic compounds ingestion and its related health issues are of concern too. These compounds are known to inhibit the uptake of iodine, hence increasing the risk of iodine deficiency disorders (e.g., endemic goiter, hypothyroidism) among consumers (15–17). With goiter rate at 10% nationwide in Ghana (18), and as high as >56% in the upper regions of the country, the contributory role of low dietary intake of iodine as a result of ingestion of residual cyanogenic compounds cannot be underestimated (17). Furthermore, cyanogenic compounds inhibit the bioavailability of sulfur-containing amino acids in protein-deficient diets (19), which is usually the case for diet for resource-poor households.

Not undervaluing the nutritional potential of conventional cassava grits, which is processed from cassava only, there is a need to explore other strategies such as compositing to improve its quality in terms of nutrients and antinutrients. To this effect, orange-fleshed sweetpotato (OFSP) could play a dual functionality as it is rich in β-carotene (20) with little or no residual cyanogenic compounds (21). OFSP is a very good source of β-carotene and other micronutrients. For instance, Apomuden, a variety in Ghana, contains between 2,100 – 5,500 μg/100 g of the provitamin A on fwb (20). A few studies have reported the presence of some antinutrients such as tannins, phytates, and oxalates in OFSP (22, 23). Other studies and reports have indicated the presence of acrylamide in sweetpotato varieties including OFSP especially when fried (24–26). These antinutrients, especially acrylamide, are injurious to human health.

Previous studies have attempted this by incorporating OFSP into cassava grits in the following ratios (cassava:OFSP): 90:10, 80:20, 70:30, 60:40, and 50:50% (27) and 90:10, 80:20, and 70:30% (11), but the samples were prepared on laboratory basis, and the residual cyanogenic compound concentration was not reported. This study therefore explored the potentials of OFSP incorporation in commercial cassava grits production and assessed the effect on β-carotene and residual cyanogenic compounds. The physicochemical and functional properties of partial substitution of cassava with OFSP to process gari were investigated. Two commonly consumed products “eba” and “soaked gari” were prepared from the various formulations and sensorially assessed.

## Materials and Methods

### Preparation of Gari From Fresh Cassava and OFSP Roots

**Figure 1:**
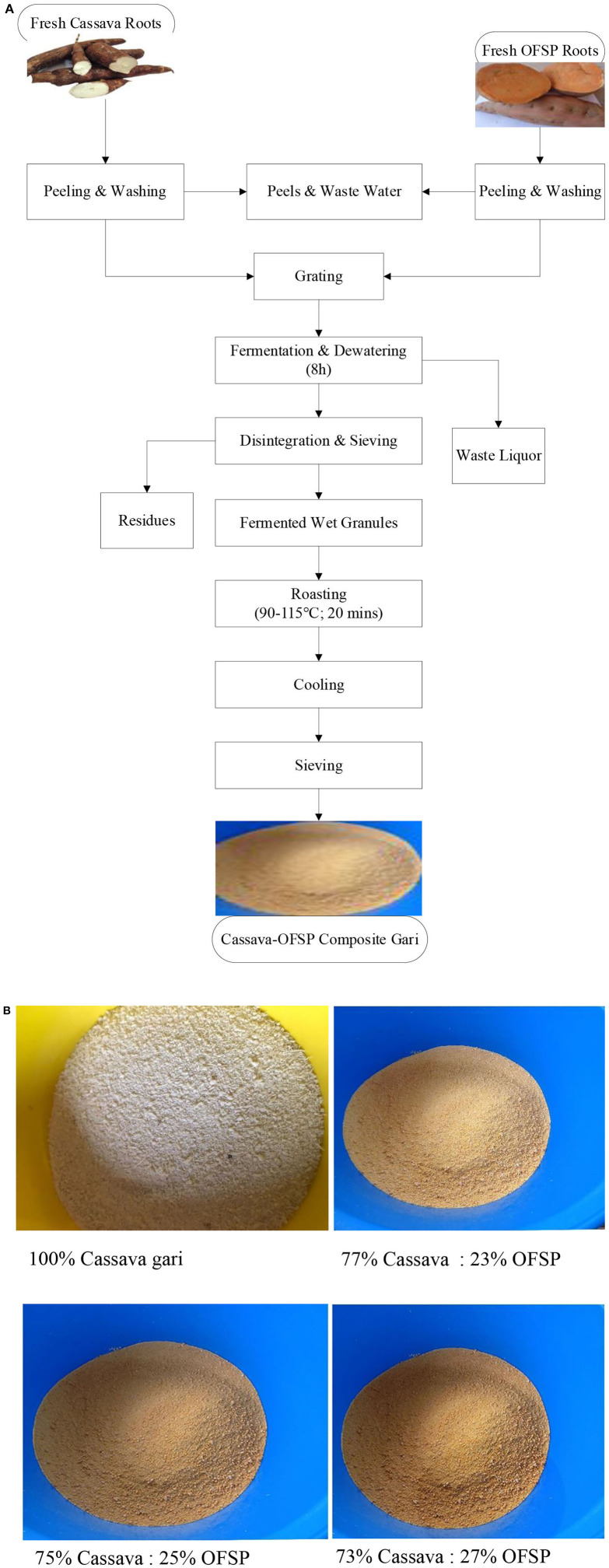
**(A)** Flowchart of the preparation of cassava OFSP composite grits. **(B)** Color of cassava-only gari and cassava-OFSP composite gari.

Gari preparation was carried out using conventional processing methods with a gari processor at Woradep village, Ho Municipal, Ghana, West Africa. The cassava (20 kg) and OFSP (10 kg) roots were separately sorted, washed, peeled, and washed again after peeling. After the second washing, the roots were mixed in ratios, wt:wt (as-is basis), of 77:23, 75:25, 73:27, and 100:0%, (cassava to OFSP) and then grated using a locally fabricated mechanized grater (Akatsi, Ghana). The substitution levels were arrived at based on earlier studies (11) that reported poor overall acceptability with OFSP substitution of more than 30%. Moreover, the local gari processor with whom the gari preparation was done has an existing recipe of 23% OFSP inclusion; hence, it was used as the base level of substitution. The resulting mash was transferred into sacks and pressed by placing weights (heavy stones) to drain off excess water. This was left overnight, ~8 h, for fermentation to occur. The fermented dough was sifted with a locally fabricated raffia mat before roasting in a locally made clay pan over a source of fire at 90–115°C for ~20 min (Figure 1A).

The gari obtained was cooled and sifted again to obtain an even particle size (Figure 1B) before packaging in black polyethylene bags placed in plastic bottles with caps. Three different batches were done to serve as replicates. The cassava-only gari from three different batches was purchased from the processor to be used as a control sample.

### Physicochemical and Functional Properties

#### pH

The pH of the gari samples was determined as described elsewhere (28) using a pH meter (Model-Basic 20 Crison Instruments, serial no. 440011, Spain).

#### Water Activity

The water activity of the gari samples was determined using a water activity meter (HBD5-ms2100Wa; Vetus Industrial Co. Ltd, China). Approximately two-thirds of the sample holder was filled with each of the grit samples. The filled sample holder was placed in the device and was sealed with masking tape and allowed to stand for 10 min before the device was turned on. After 5 min, the water activity readings were taken.

#### Water Absorption Capacity

The water absorption capacity (WAC) of each of the gari samples was determined according to the method of Badmus et al. (29).

#### Swelling Index

The method of Onwuka (30) was used to determine the swelling index (SI) of each of the samples.

#### Color Determination

The color of the gari samples was determined with a handheld Chroma Meter (Konica Minolta CR-410) as described elsewhere (31).

#### Proximate Analysis

The Official Methods of Analysis of the Association of Official Analytical Chemists (AOAC) International (32) was used to determine the moisture (AOAC 925.10), crude protein (AOAC 960.52), ash (923.03), and crude fat (AOAC 922.06). Total carbohydrate was determined by difference. Thus, total carbohydrate = 100 – [moisture + crude protein + ash + crude fat].

#### β-Carotene

The samples were analyzed for the β-carotene content following the protocols reported by Mackinney (33) using a spectrophotometer (UV/VIS Excellence UV5; Mettler Toledo, Switzerland). β-Carotene was converted to vitamin A using the relation: retinol activity equivalents (RAEs) using 1 RAE = 12 μg β-carotene (34).

#### HCN Determination

The cyanide content of the grit samples was determined using the method described elsewhere (35).

### Product Preparation and Sensory Evaluation

“Eba” is a native Nigerian staple prepared with gari. “Eba” was prepared as reported elsewhere (36) with slight modification. In the modification, 750 mL of water was brought to boil for 2 min. Approximately 500 g of the OFSP-cassava composite gari (Figure 1B) and 5 g of salt were then gradually added to the boiling water and continuously stirred with a stirring rod to obtain a smooth and homogeneous dough. The sticky leathery dough was then molded into balls. The prepared eba was then stored in an ice chest to maintain temperature before the sensory assessment. One hundred untrained panelists who were familiar with the product scored the product using a 5-point hedonic scale (1 = extremely dislike to 5 = extremely like).

The “soaked gari” was prepared as described by Teeken et al. (37). The panelists were presented with 5 g of gari, 2.5 g of sugar, and 10 mL of cold water to use to prepare as they would on their own. They assessed the product for color, aroma, taste, texture, and overall acceptability using a 5-point hedonic scale: 1 = extremely dislike to 5 = extremely like.

### Statistical Analyses

Data were analyzed using a one-way analysis of variance (ANOVA) procedure. The Tukey studentized range test was used to compare differences between means when the ANOVA result was significant (*p* < 0.05). Association between β-carotene, HCN, and color parameters such as L*, a*, and b* was tested using Pearson correlation. A one-sample *t* test was carried out to compare the mean HCN of samples to the codex maximum threshold. All statistical procedures were done using Minitab software version 16.

## Results and Discussion

### Physicochemical Properties of Cassava OFSP Composited Gari

**Figure 2:**
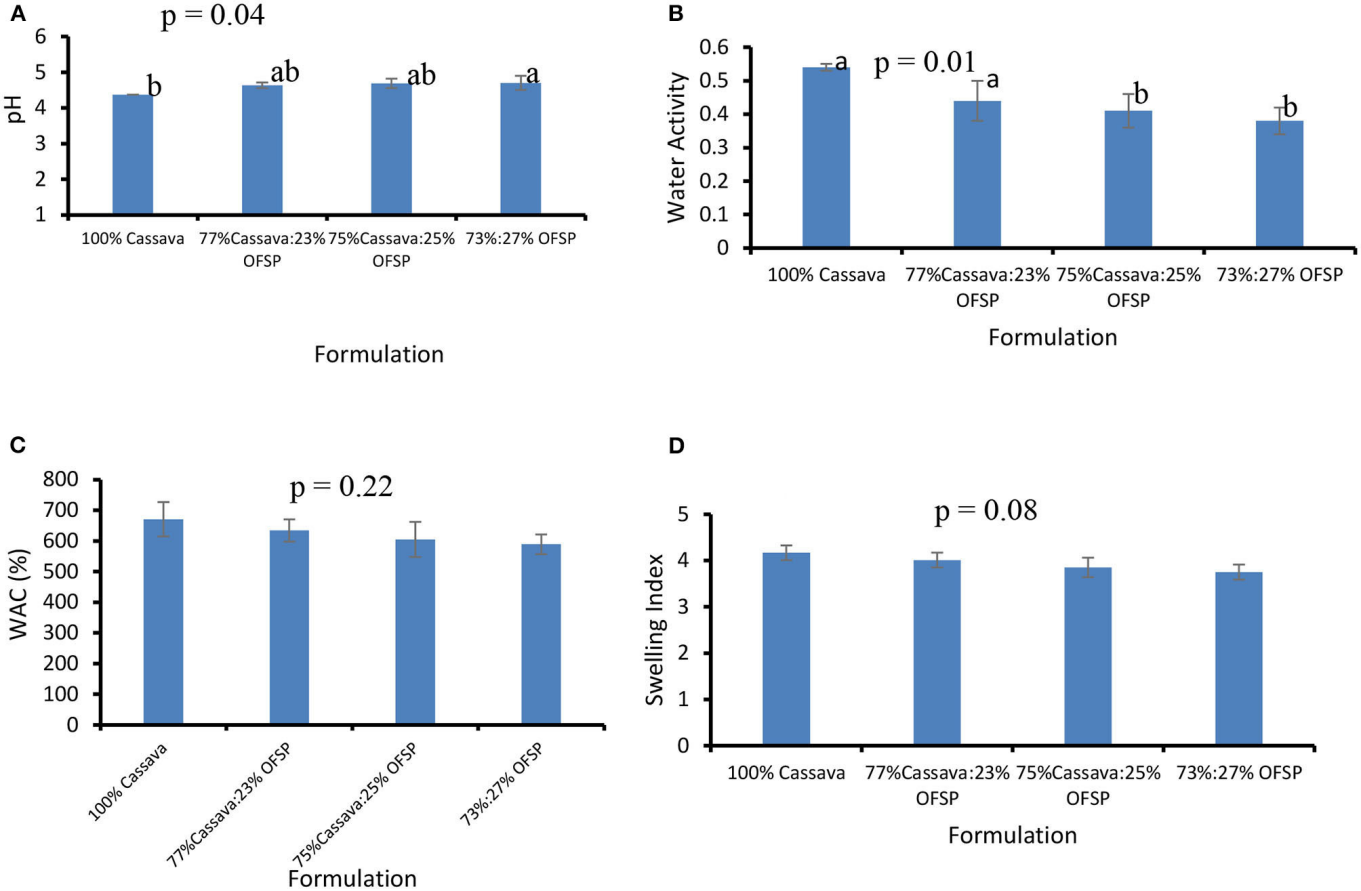
Physicochemical properties: pH **(A)**, water activity **(B)**, WAC **(C)**, and swelling index **(D)** of cassava OFSP composite grits. Bar values are means ± standard deviation. WAC, water absorption capacity.

The 100% cassava gari had a significantly (*p* = 0.04; 4.37 vs. 4.70) lower pH compared to the blend with the highest level of OFSP (73% cassava:27% OFSP) as presented in Figure 2A. The pH of the samples slightly increased with the incorporation levels of OFSP. This could be due to declined carbohydrate availability and thus decreased level of fermentable substrates (38). The pH of all the formulations was generally low because of the fermentation process. The pH of gari is a function of fermentation and an indicator of its storage life. The lower the pH, the better the storage life as it inhibits microbial activity. Values obtained in this study agree with those reported by other studies (5, 21).

Figure 2B presents data on the water activity of the various formulations. The 100% cassava formulation had a significantly (*p* = 0.01) higher water activity relative to the composited samples. The water activity ranged from 0.54 in the 100% cassava to 0.38 in the 73% cassava:27% OFSP. The incorporation of OFSP resulted in declined water activity. This could be due to the presence of sugar molecules in the OFSP that could absorb the water molecules, thereby making them unavailable, hence the lower water activity. Water-soluble compounds, such as sugar and salt gums, have long been recognized as possessing a water-binding effect to reduce water activity (39). Water activity is an important factor as far as the storage life of food products is concerned. Lower water activity translates to longer shelf life and *vice versa*.

The ability of the gari to swell at least three times its initial volume is what consumers consider as good gari (40) as this gives a higher volume and a sense of satiation. In this study, the WAC and SI of the cassava OFSP-composited gari at all levels of inclusion were like the 100% cassava gari. The WAC ranged from 589% for 73% cassava:27% OFSP to 670.9% in the 100% cassava (Figure 2C). The SIs of the 100% cassava and 73% cassava:27% OFSP were 4.2 and 3.8, respectively (Figure 2D). Similar WAC and SI values were reported earlier for cassava sweetpotato composited gari (21).

### Color of Cassava OFSP Composite Gari

Color is one of the important quality criteria that influence consumers' food choices (41). Consumers in Ghana consider the whiteness of gari as a measure of purity. However, yellow hued gari is priced higher because of the belief that it has added health-promoting nutrients from food color or red palm oil (11). In this study, the increases in the amount of OFSP in the composited blend decreased the brightness of the gari significantly (*p* < 0.001; L* = 69.26) by almost 1.2 times (Table 1). Expectedly, the degree of redness (a*) and yellowness (b*) of the grits increased significantly (*p* < 0.001) with the incorporation level of OFSP. The reduction of the whiteness (L*) level and the increase in the redness (a*), yellowness (b*), and color intensity (C*) of the composited grits are attributable to the orange color of the OFSP incorporated. The findings corroborate Abano et al. (11) study. Furthermore, the increased redness in the OFSP-based gari could partly be attributed to caramelization of the high sucrose in the OFSP (11). The total color change (ΔE*) increased with increased OFSP inclusion levels following a reduction in the lightness (L*) and an increase in redness (a*) and yellowness (b*) of the samples as imparted by the OFSP. The relatively higher a* and b* values of the OFSP-based grits could be an indication of the presence of health-promoting bioactive compounds including β-carotene.

**Table 1:** Color properties of cassava-OFSP composited gari.

	**Color parameter**				
**Formulation**	L^*^	a^*^	b^*^	C^*^	ΔE^*^
100% Cassava	83.99 ± 0.13^a^	0.93 ± 0.04^a^	16.35 ± 0.20^a^	17.22 ± 0.23^a^	85.58 ± 0.00^a^
77% Cassava:23% OFSP	73.17 ± 0.41^b^	5.49 ± 0.20^b^	25.34 ± 0.04^b^	55.58 ± 2.15^b^	77.63 ± 0.44^b^
75% Cassava:25% OFSP	71.34 ± 0.170^c^	6.89 ± 0.09^c^	27.39 ± 0.15^c^	74.96 ± 1.39^c^	76.73 ± 0.18^c^
73% Cassava:27% OFSP	69.26 ± 0.07^d^	7.74 ± 0.21^d^	28.62 ± 0.55^d^	88.51 ± 3.75^d^	75.34 ± 0.14^d^
***p*****-value**	**<0.001**	**<0.001**	**<0.001**	**<0.001**	**<0.001**

* *Values are means ± SD (n = 3). Means in the same column with different superscripts are significantly different (p < 0.05)*.

### Proximate Analysis

The proximate parameters analyzed showed no significant differences (*p* > 0.05) except for crude ash (Table 2). It is noteworthy that the moisture content of all samples was lower (Table 2) than the maximum reference level, 12% for gari (42), an important indicator for good shelf life.

**Table 2:** Proximate analysis of OFSP-cassava composited gari (on dry matter basis).

**Formulation**	**Proximate parameters (%)**				
	**Moisture**	**Crude ash**	**Crude protein**	**Crude fat**	**Total CHO**
100% Cassava	5.42 ± 0.15	1.28 ± 0.04^b^	1.37 ± 0.10	0.25 ± 0.09	91.68 ± 0.16
77% Cassava:23% OFSP	4.07 ± 2.75	1.83 ± 0.08^a^	1.49 ± 0.18	0.25 ± 0.02	92.26 ± 2.34
75% Cassava:25% OFSP	3.78 ± 0.24	1.92 ± 0.07^a^	1.52 ± 0.05	1.60 ± 0.99	91.18 ± 0.69
73% Cassava:27% OFSP	3.39 ± 0.60	1.93 ± 0.03^a^	1.58 ± 0.15	2.93 ± 4.25	90.28 ± 3.70
***p*****-value**	**0.38**	**<0.01**	**0.33**	**0.43**	**0.75**

*Values are means ± SD (n = 3). Means in the same column with different superscripts are significantly different (p < 0.05)*.

The crude ash content of the cassava OFSP composite gari was significantly (*p* < 0.01) higher than the 100% cassava gari. The data further showed that the total ash content increased with increasing levels of OFSP with the 73% cassava:27% OFSP composite gari recording almost 1.5 times higher than the 100% cassava gari. The findings corroborate previous works by Ojo and Akande (27) and Karim et al. (21), who reported a significant increase in ash content with an increased inclusion level of OFSP compared to 100% cassava gari. The crude ash content depicts the total mineral content in food, indicating higher mineral content in the composited gari.

Although no significant (*p* = 0.33) differences were observed among formulations, the crude protein increased with increasing levels of OFSP (Table 2), similar to the findings of Ojo and Akande (27). The increase in protein content of the cassava OFSP composite gari with increased levels of OFSP could be attributed to the relatively higher protein content in sweetpotato compared to cassava (43).

Similarly, there were no marked (*p* = 0.43) differences among the formulations in terms of crude fat content ranging from 0.25 to 2.93%. However, the crude fat content increased as the OFSP inclusion level increased and corroborated the findings of Ojo and Akande (27). The increase in the crude fat content of cassava OFSP composite gari could be due to the relatively higher fat content in OFSP roots compared to cassava and other root and tuber crops as reported elsewhere (44).

The total carbohydrate content did not vary significantly (*p* = 0.75) among the formulations (Table 3). Expectedly, the total carbohydrate content of the composite gari decreased with the increased addition of OFSP. This was largely because OFSP cultivars are generally lower in carbohydrates compared to cassava.

**Table 3:** Mean HCN concentration of samples compared to the 2-mg/kg threshold.

**Formulation**	**HCN (mg/kg)**	**SD**	***t*-value**	***p*-value**
100% Cassava	1.71	0.1287	−3.95	0.060
77% Cassava:23% OFSP	0.89	0.2240	−8.58	0.013
75% Cassava:25% OFSP	0.59	0.1285	−18.95	0.003
73% Cassava:27% OFSP	0.37	0.1285	−21.96	0.002

### β-Carotene of Cassava OFSP–Based Gari

**Figure 3:**
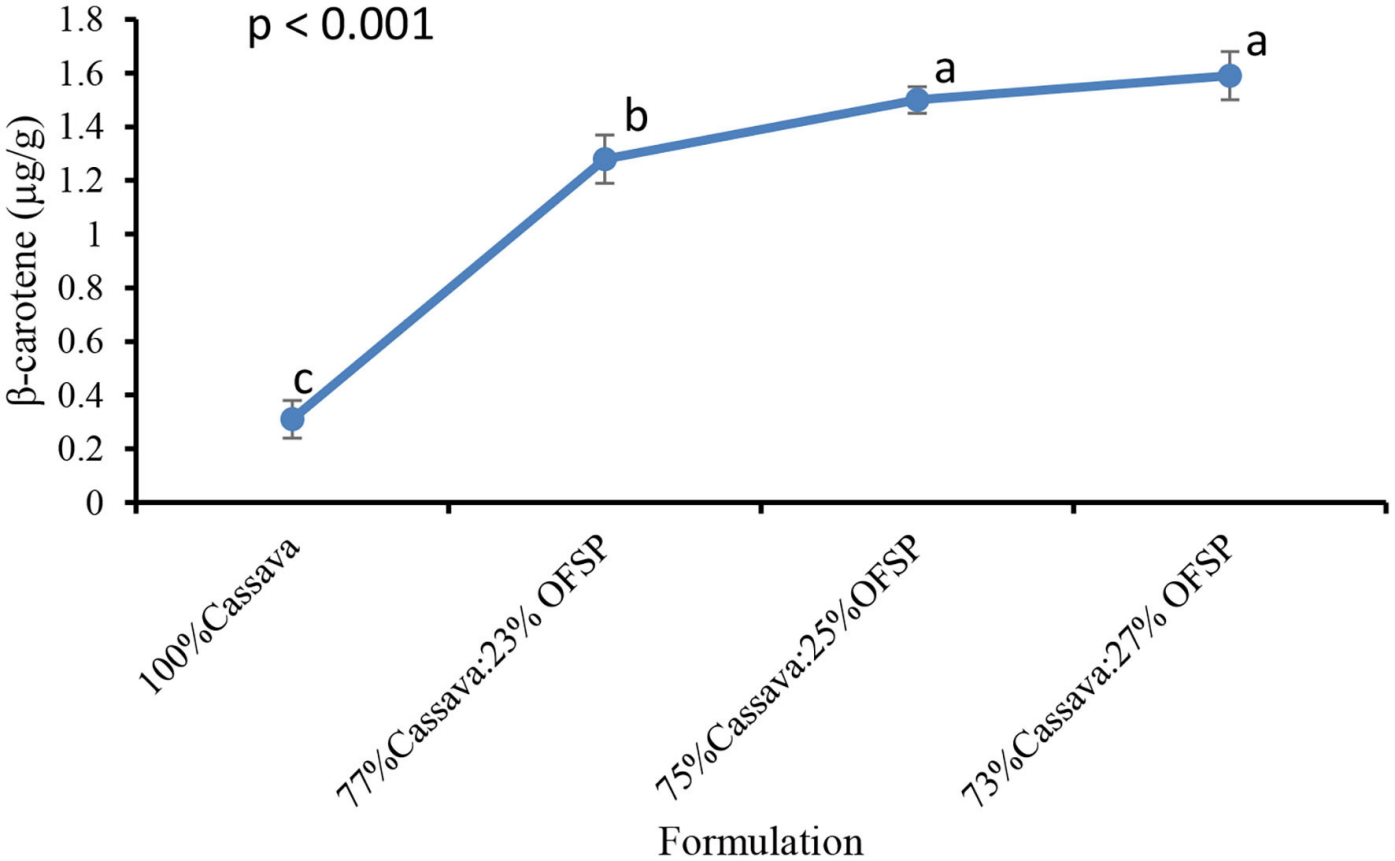
Effect of OFSP incorporation on the β-carotene content of gari. Bars are means ± SD (*n* = 3). Means with different letters are significantly different (*p* < 0.05).

The β-carotene content of the cassava OFSP composite gari was directly related to the proportion of OFSP incorporated (Figure 3). The β-carotene content of the 73% cassava:27% OFSP blend was ~5 times significantly (*p* < 0.001) higher than the 100% cassava gari. This result corroborates with Abano et al. (11), who reported higher levels of β-carotene in cassava OFSP composite gari as the OFSP concentration increased.

A test of association between the β-carotene content of the gari samples showed almost a perfect positive correlation (*r* = 0.99) with redness (a*) and yellowness (b*) values and *vice versa* with whiteness. This association between the β-carotene content and redness and yellowness was expected as red- and yellow-pigmented food substances are associated with high carotenoid content.

Based on the levels of the provitamin A and its conversion ratio to the daily Recommended Dietary Allowance (RDA) of vitamin A and assuming an estimated daily gari intake of 551 g/day by Ghanaian adults, the 27% OFSP-based gari will, respectively meet almost 10 and 6% of the RDA of pregnant women (770 μg RAE/day) and lactating mothers (1,300 μg RAE/day) (34). The 100% cassava gari would only meet 2% and 1% of the RDA of pregnant women and lactating mothers, respectively. The consumption of the OFSP-cassava composite grits may be a useful tool in reducing VAD and its related problems in Ghana.

### Hydrogen Cyanide Content of Cassava OFSP Composite Gari

**Figure 4:**
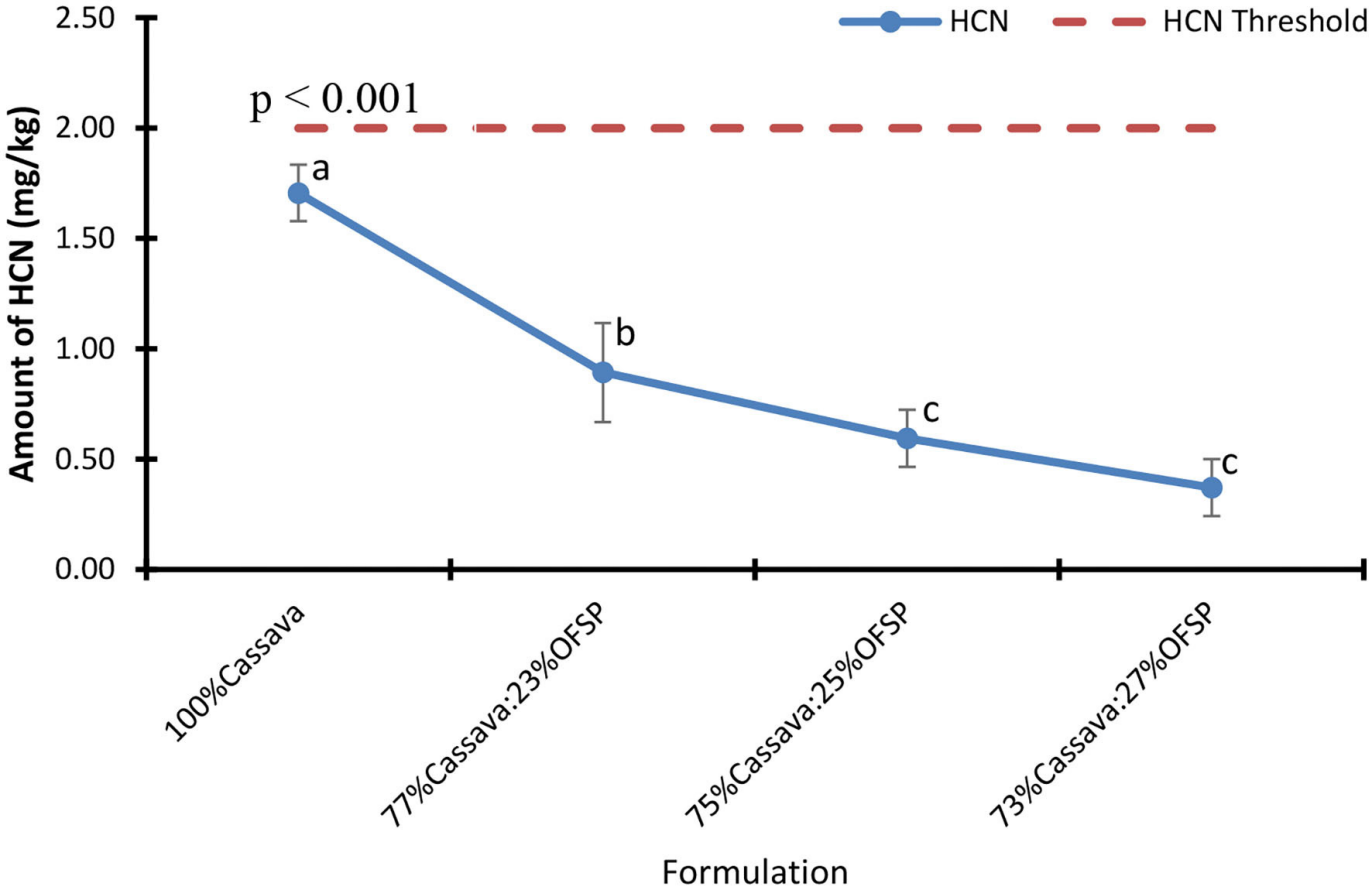
HCN content of gari. Bars are means ± SD (*n* = 3). Means with different letters are significantly different (*p* < 0.05).

The HCN content of the 100% cassava gari was significantly (*p* < 0.001) higher than the OFSP-based gari, almost two and five times higher than that of the 23 and 27% OFSP-composited gari, respectively. It was observed that the HCN content decreased with increased levels of OFSP (Figure 4). A similar trend was observed when white-fleshed sweetpotato was incorporated (21). The significant reduction in HCN characterized by the OFSP-based gari could be attributed to the inclusion of OFSP that is reported to low in HCN (20).

A correlational analysis revealed that HCN had a strong positive (*r* = 0.96, *p* = 0.001) relationship with whiteness values but a strong inverse relationship with β-carotene (*r* = −0.96, *p* = 0.001), redness (*r* = −0.97, *p* = 0.001), and yellowness (*r* = −0.97, *p* = 0.001) values. This result is expected because the OFSP has no HCN, and as there is a reduction in cassava with HCN and an increase in OFSP, the HCN level will reduce, and β-carotene will increase.

A one-sample *t*-test showed no significant (*p* = 0.06) difference between the mean HCN content of 100% and the Codex (45) permissible level of 2 mg/kg. However, the OFSP-based gari was significantly (*p* < 0.05) lower than the Codex maximum threshold (Table 3). The HCN content of the highest OFSP incorporation (27% OFSP) was almost 5.4 times lower than the maximum threshold of 2.0 mg/kg. The data thus suggest that frequent consumption of 100% cassava gari could cause HCN problems over a short time as compared to the cassava OFSP-composited gari. Cyanogenic glycosides generally occur when plant materials are crushed either during the consumption or processing of a food crop. The subsequent hydrolysis of these compounds results in the formation of HCN (9). Dietary exposure to high levels of HCN in food could cause acute cyanide poisoning. Although the human body has a detoxification mechanism for cyanide, it can only do this efficiently at lower levels (35).

### Consumer Acceptability of “Eba” and “Soaked Gari”

**Figure 5:**
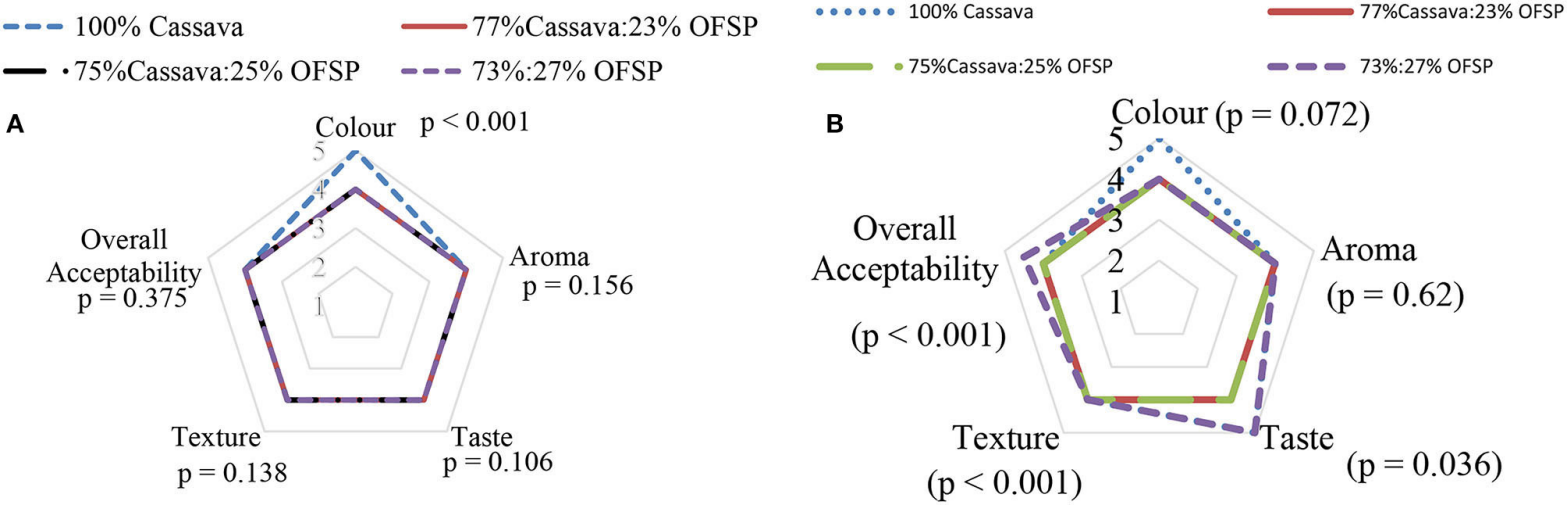
**(A)** Consumer acceptability score of “eba” prepared from OFSP-composited gari. **(B)** Consumer acceptability score of “soaked gari” prepared from OFSP-composited gari. Values are medians, *n* = 100.

Apart from color for “eba,” that the 100% cassava gari was significantly (*p* < 0.001) preferred, all the other attributes' scoring indicates that the OFSP-composited gari will generally be accepted by Ghanaian consumers when used for “eba” or “soaked gari” (Figure 5A). This was not surprising because most Ghanaian consumers perceive that the acceptable color for gari is white and nothing more. Promotional campaigns need to be intensified to highlight the importance of consuming brightly colored foods in order to address the relatively lower preference for color.

The consumer acceptability study of the two products prepared from the various formulations of OFSP showed a very good consumer acceptability (score ≥4) for both products in terms of all the sensory attributes evaluated.

For a taste for “soaked gari” (a mixture of gari, sugar, and water), the 73% cassava:27% OFSP blend and the 100% cassava gari recorded significantly (*p* = 0.036) higher scores for “soaked gari” (Figure 5B).

This is an indication that the OFSP-based gari has great potential in the Ghanaian market as taste is an important driver of consumers' choice of a product (46). The finding implies that consumer acceptability of the OFSP-based gari prepared into “soaked gari” has similar sensory characteristics as the conventional gari and could be accepted in the Ghanaian consumers.

## Conclusion

OFSP can be composited with cassava up to 27% incorporation to process gari. This composited sample has similar physicochemical characteristics (pH, WAC, and SI) and sensorial properties compared to the 100% cassava only except for the color for “eba,” which was ranked higher than the composited gari. Furthermore, OFSP-composited gari has a significantly higher dietary β-carotene and lower HCN compared with the 100% cassava-only gari. Thus, the composited gari could be more beneficial in addressing VAD in Ghana as 100% white-fleshed cassava gari contains minimal or no provitamin A necessary to address VAD.

Future studies on the digestibility of the composited gari and the determination of antinutrients such as acrylamide, which is associated with heat processing of OFSP, should also be considered. Besides, a profitability analysis on producing the biofortified gari, relative to the standard 100% cassava gari, would also be a critical factor with potential for adoption.

## Data Availability Statement

The original contributions presented in the study are included in the article/supplementary material, further inquiries can be directed to the corresponding author.

## Author Contributions

RA and FA: experimental design and supervision. RA, MA, and TA: sampling, analytical determinations, and manuscript draft preparation. RA, MA, TA, and FA: statistical analysis and data interpretation and revision of the draft. All authors contributed to the article and approved the submitted version.

## Conflict of Interest

The authors declare that the research was conducted in the absence of any commercial or financial relationships that could be construed as a potential conflict of interest.
